# Clinical significance of multiparametric MRI and PSA density as predictors of residual tumor (pT0) following radical prostatectomy for T1a-T1b (incidental) prostate cancer

**DOI:** 10.1371/journal.pone.0210037

**Published:** 2018-12-28

**Authors:** Doo Yong Chung, Hyeok Jun Goh, Dong Hoon Koh, Min Seok Kim, Jong Soo Lee, Won Sik Jang, Young Deuk Choi

**Affiliations:** Department of Urology, Urological Science Institute, Yonsei University College of Medicine, Seoul, Korea; University of South Alabama Mitchell Cancer Institute, UNITED STATES

## Abstract

**Purpose:**

The aim of this study was to evaluate predictors of residual tumor and clinical prognosis in T1a-T1b (incidental) prostate cancer by analysis of specimens from men undergoing surgery for benign prostatic hyperplasia.

**Materials and methods:**

We retrospectively reviewed medical records of incidental prostate cancer patients who had undergone radical prostatectomy. Patients whose tumor statuses were further confirmed by prostate biopsy, or who had used androgen deprivation therapy before radical prostatectomy, were excluded. Clinical and pathological parameters were analyzed to evaluate residual tumor and clinical prognosis. We used univariate and multivariate logistic regression analyses, as well as receiver operator characteristics, to predict residual tumor (pT0).

**Results:**

The final analysis included 95 patients. Among these patients, 67 (70.53%) exhibited residual tumor, whereas 28 (29.47%) did not (pT0). Pathology findings showed that 44 (65.67%), 16 (23.88%), and 7 patients (10.45%) exhibited Gleason scores of G6, G7, and ≥G8, respectively. Fifty-seven and 10 patients exhibited pathologic T stages T2 and T3, respectively. Mean follow-up duration was 70.26 (±34.67) months. Biochemical recurrence was observed in 11 patients; none were pT0 patients. Multivariate logistic regression showed that low prostate-specific antigen density after benign prostatic hyperplasia surgery and invisible lesion on multiparametric magnetic resonance imaging were significantly associated with pT0. Additionally, a combination of these factors showed an increase in the diagnostic accuracy of pT0, compared with mpMRI alone (AUC 0.805, 0.767, respectively); this combination showed sensitivity, specificity, and positive predictive values of 71.6%, 89.3%, and 94.1%, respectively.

**Conclusion:**

Our results suggest that patients with incidental prostate cancer who have both prostate-specific antigen density ≤0.08 after benign prostatic hyperplasia surgery as well as invisible cancer lesion on multiparametric magnetic resonance imaging should be considered for active surveillance.

## Introduction

Prostate cancer (PCa) is one of the most prevalent cancers worldwide; moreover, it exhibits the highest incidence among all cancers in males in the United States, especially in elderly individuals [1]. As PCa screening through measurement of prostate-specific antigen (PSA) levels has become more widespread, the proportion of PCa patients presenting with low-risk factors has also increased [2]. According to the European Association of Urology guidelines, an extended 12-core systematic transrectal ultrasound (TRUS)-guided biopsy should be performed for patients with an elevated PSA level; this is endorsed as the optimal biopsy method [3]. Therefore, in the PSA era, most cases of PCa are found by prostate biopsy; the number of prostate cancers found by benign prostatic hyperplasia (BPH) surgery is reportedly decreasing [4]. However, during BPH surgery, PCa was found incidentally in 5–13% of patients who did not have a prior diagnosis [5,6]. According to the TNM staging system, the presence of incidental PCa (IPCa) in less than 5% of resected prostate tissue is classified as clinical stage T1a; its presence in more than 5% of resected tissue is classified as T1b. Although most cases of IPCa are considered clinically insignificant, recent studies have suggested that in some cases, the prognosis may become more unfavorable [7]. Therefore, controversies exist regarding the most appropriate management for patients diagnosed with IPCa. Several guidelines suggest radical prostatectomy (RP) as treatment for patients with a life expectancy of more than 10 years [3].

Notably, the probability of finding no residual cancer (pT0) among patients with IPCa who undergo RP has been reported in several studies [5,8–10]. In patients with IPCa, the vanishing cancer phenomenon is more likely to be related to the presence of a small cancer that can be entirely removed during initial surgery [11]. If pT0 can be predicted by preoperative assessment of patients with IPCa, overtreatment may be avoided. Previous studies have used factors such as PSA and Gleason score (GS) to evaluate the significance of IPCa. Recently, there has been a rapid increase in the use of multiparametric magnetic resonance imaging (mpMRI) for diagnosis and staging of PCa. Therefore, in this study, we evaluated whether pT0 could be predicted in patients with IPCa by using preoperative diagnostic tools, including mpMRI.

## Materials and methods

### Study design and patients

We retrospectively reviewed the clinical and pathological data of 107 individuals with PCa who underwent BPH surgery before RP at our institution between June 2006 and December 2016; patients whose tumor statuses were further confirmed by prostate biopsy during surgery for BPH, or who had used androgen deprivation therapy before RP, were excluded. Therefore, 95 patients were included in this analysis; all included patients underwent mpMRI before RP. All images were retrospectively reviewed by three experienced uroradiologists who were blinded to pathologic results; they conducted a consensus review of the mpMRI images of all patients. The mpMRI images included standardized criteria for Likert scoring of multiparametric sequences using a 3.0-T MRI system (Intera Achieva 3.0-T, Phillips Medical System, Best, The Netherlands) [12]. From 2006 to 2009, mpMRI included T1-weighted [T1W] and T2-weighted [T2W] imaging, as well as dynamic contrast-enhanced imaging [DCE]. Diffusion-weighted imaging [DWI] and the apparent-diffusion coefficient [ADC] have been added since 2010. In mpMRI, suspicious lesions were graded 1–5 by using a scoring system established by the Prostate Imaging Reporting and Data System (PI-RADS) [13,14]. Negative MRI findings were defined as the absence of grade 3 or higher regions of interest (ROIs) [15,16]. We also excluded patients with limitations with regard to interpretation by the radiologists due to mpMRI without DWI and ADC. PI-RADS^V2^ was used as a standard when the image was reviewed again by radiologists. Clinical characteristics of these patients included age, body mass index, PSA before and after BPH surgery, and prostate volume (measured by TRUS) before and after BPH surgery [17]. In addition, Gleason score (GS), resection volume, and tumor volume following BPH surgery, as well as pathologic characteristics of specimens following RP, were obtained. All pathologic diagnoses were performed by expert pathologists. Finally, TNM stage was determined in accordance with the 8th edition of the American Joint Committee on Cancer TNM staging system.

### Follow-up

Postoperative PSA follow-up was performed monthly for the first 6 months, every 3 months for the second year, and every 6 months thereafter. Biochemical recurrence (BCR) was defined as any two consecutive increases in serum PSA ≥0.2 ng/ml following RP [18]. BCR-free survival was defined as the time from RP to BCR. The follow-up period was calculated from the date of RP to the date of the last known contact with the patient.

## Research involving human participants and/or animals

All procedures performed in studies involving human participants were conducted in accordance with the ethical standards of the institutional and/or national research committee and with the 1964 Helsinki declaration and its later amendments or comparable ethical standards. For this type of study formal consent is not required. Data were collected after approval from the Institutional Review Board at Yonsei University College of Medicine (protocol number 4-2018-0669).

## Informed consent

Informed consent was not required from individual participants included in the study due to its retrospective design involving review of medical records.

### Statistical analysis

We compared clinical and pathological characteristics between groups by using Mann–Whitney U tests for continuous data and χ^2^ tests for dichotomous variables. Univariate and multivariate logistic regression analysis were performed to assess the association between baseline parameters and residual cancer. Significant variables from univariate analysis were included in the multivariate analysis. Moreover, the Kaplan-Meier method, combined with log-rank tests, was performed to estimate and compare oncologic outcomes with respect to pT0. Receiver operator characteristic (ROC) curve analysis was performed to determine the optimal cut-off value via the area under the curve (AUC). Comparisons where *p* < 0.05 were considered statistically significant. These statistical analyses were performed with SPSS Statistics software, version 23.0 (IBM, Armonk, NY, USA). In addition, assessments of sensitivity, specificity, positive predictive value (PPV), and negative predictive value (NPV) with 95% confidence intervals were performed with Medcalc (version 18.3; Mariakerke, Belgium).

## Results

### Patient and disease characteristics

A total of 95 incidental PCa patients were included. At the time of surgery for BPH, patients were classified as stage T1a (n = 49) or T1b (n = 46), in accordance with the 8th edition of the American Joint Committee on Cancer TNM staging system. The mean age for all patients was 67.31±4.93 years. The mean prostate volume, as measured by TRUS, was 39.88±16.97 ml; the mean PSA and PSA density values were 4.45±3.63 ng/ml and 0.13±0.14 ng/ml^2^, respectively. The mean resection volume was 11.17±10.35 ml; the mean PSA and PSA density values after BPH surgery were 1.58±1.40 ng/ml and 0.06±0.06 ng/ml^2^. Among specimens following BPH surgery, 75 exhibited GS 6 (76.1%), 13 exhibited GS 7 (17.4%), and 7 exhibited GS ≥8 (6.6%). Forty-nine patients (51.6%) showed no suspicious lesions during MRI performed to evaluate the presence of PCa. Of the 46 patients (48.4%) with suspicious lesions, 33 showed lesions in the peripheral zone, 9 showed lesions in the transitional zone, and 4 showed lesions in both the peripheral and transitional zones.

The median follow-up from RP was 68.37±41.83 months. Among specimens following RP, 67 (70.53%) exhibited residual tumor and 28 (29.47%) did not (pT0). When dividing the two groups on the basis of residual tumor, there were significant differences between the two groups in PSA after BPH surgery, PSA density before and after BPH, and suspicious lesions in MRI. T stage, according to BPH surgery and PSA before BPH surgery, did not significantly differ between the two groups (Table 1).

**Table 1:** Baseline patient characteristics.

Variable		Total		Residual tumor		No_Residual tumor		p value
		N = 95	SD	N = 67 (70.53%)	SD	N = 28 (29.47%)	SD	
**Age, years**		67.31	4.93	67.67	4.77	66.43	5.28	0.264
**BMI, kg/m**^**2**^		24.12	2.68	23.58	2.48	25.42	2.75	0.002
**PSA level before BPH surgery, ng/ml**		4.45	3.63	4.79	3.87	3.64	2.89	0.163
**PSA density before BPH surgery, ng/ml**^**2**^		0.13	0.14	0.15	0.16	0.08	0.06	0.005
**PSA level after BPH surgery, ng/ml**		1.58	1.40	1.82	1.47	1.01	1.00	0.003
**PSA density after BPH surgery, ng/ml**^**2**^		0.06	0.06	0.07	0.06	0.03	0.02	<0.001
**Prostate volume, ml**		39.88	16.97	38.19	15.79	43.91	19.22	0.135
**Resection volume, ml**		11.17	10.35	10.47	10.04	12.98	11.05	0.273
**Duration between operations, days**		146.19	193.76	151.12	214.80	134.39	133.11	0.703
**Suspicious lesion on MRI**		N	%	N	%	N	%	<0.001
**Yes**		46.00	48.42	43.00	64.18	3.00	10.71	
**No**		49.00	51.58	24.00	35.82	25.00	89.29	
**Gleason score**		N	%	N	%	N	%	0.545
**6 (ISUP G1)**		75.00	78.95	51.00	76.12	24.00	85.71	
**7**	**ISUP G2**	4.00	9.47	4.00	5.97	0.00	0.00	
	**ISUP G3**	9.00	4.21	7.00	10.45	2.00	7.14	
**≥8 (ISUP G4)**		7.00	7.37	5.00	7.46	2.00	7.14	
**Stage**		N	%	N	%	N	%	0.084
**T1a**		49.00	51.58	31.00	46.27	18.00	64.29	
**T1b**		46.00	51.58	36.00	53.73	10.00	35.71	
**FU duration after RP, months**		68.37	41.83	71.54	36.16	70.26	34.67	0.874

BMI, body mass index; PSA, prostate-specific antigen; BPH, benign prostatic hyperplasia; ISUP, International Society of Urological Pathologists; RP, radical prostatectomy; FU, follow-up

### Oncologic outcomes following RP

**Fig 1:**
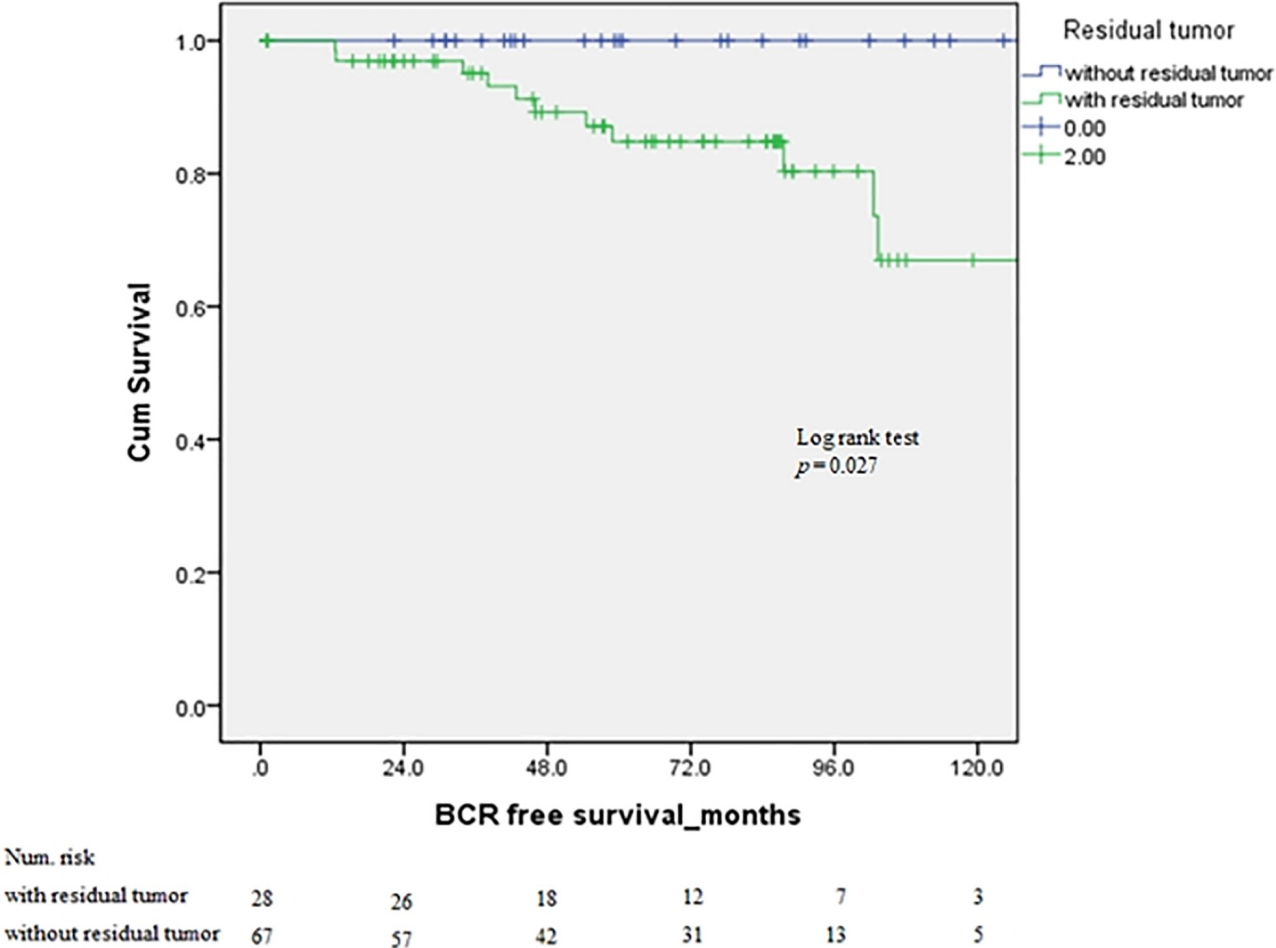
Kaplan-Meier curves for biochemical recurrence (BCR)-free survival in patients according to residual tumor (pT0).

Among 67 RP specimens (excluding those with pT0), 44 exhibited GS 6 (65.7%) and 16 exhibited GS 7 (23.9%). Furthermore, GS ≥8 was present in 7 (10.4%). The median tumor volume of specimens following RP was 0.76±1.1 ml. Pathologic stage ≥T3 was recorded in 10 cases (14.9%). Extracapsular extension (ECE) was present in 10 cases (14.9%); surgical margins were involved in 4 (6.0%). Invaded seminal vesicles were observed in 1 case (1.5%). Perineural invasion was reported in 1 case (1.5%); lymphovascular invasion (LVI) and lymph node metastasis were not reported. During the follow-up period, BCR was not observed in pT0 patients; in the residual PCa group, BCR was observed in 11 cases (16.4%). Furthermore, there were no cancer-specific deaths during the observation period (Table 2). Additionally, Kaplan-Meier curves showed a significant increase in BCR-free survival in the pT0 group (log-rank test, *p* = 0.027) (Fig 1). Univariate and multivariate Cox regression analyses were performed with each clinical parameter for BCR in patients with residual cancer. In these analyses, GS ≥8 (HR 18.235, *p* = 0.001) and pathologic T stage ≥ T3 (HR 13.899, *p* < 0.001) were independent prognostic factors for BCR. In contrast, PSA, PSA density, and T1a or T1b were not statistically different.

**Table 2:** Characteristics of patients with residual tumor following radical prostatectomy.

Variable		Total	
		N = 67	
**Pathologic Gleason score**		N	%
**6 (ISUP G1)**		44	65.67
**7**	**(ISUP G2)**	13	19.40
	**(ISUP G3)**	3	4.48
**≥8 (ISUP G4)**		7	10.45
**Tumor volume, ml**		0.76	1.10
**Pathologic T stage**		N	%
**≤T2**		57	85.07
**≥T3**		10	14.93
**ECE**		N	%
		10	14.93
**SVI**		N	%
		1	1.49
**PSM**		N	%
		4	5.97
**LVI**		N	%
		0	0.00
**PNI**		N	%
		1	1.49
**BCR**		N	%
		11.00	16.42

ISUP, International Society of Urological Pathologists; ECE, extracapsular extension; SVI, seminal vesicle invasion; LVI, lymphovascular invasion; PSM, positive surgical margin; PNI, perineural invasion; BCR, biochemical recurrence

### Preoperative factors in relation to pT0 PCa

In this study, we used univariate and multivariate logistic regression analyses to identify predictors associated with pT0 PCa. In these analyses, PSA density after BPH surgery (Odds ratio [OR]: 0.684, 95% confidence interval [CI]: 0.469–0.997, *p* = 0.048) and suspicious lesion on mpMRI (OR: 11.827, 95% CI: 3.013–45.073, *p* = 0.001) constituted independent predictors of the presence of residual cancer at RP in both univariate and multivariate models. After BPH surgery, invisible lesion on mpMRI and low PSA density showed a significant correlation with pT0 (Table 3).

**Table 3:** Univariate and multivariate analyses of factors associated with residual tumor (pT0).

Variable		Univariate		Multivariate	
		OR (95% CI)	p value	OR (95% CI)	p value
**Age, year**		0.949 (0.866–1.040)	0.263		
**PSA level before BPH surgery, ng/ml**		0.905 (0.785–1.043)	0.167		
**PSA density before BPH surgery, ng/ml**^**2**^		0.942 (0.887–1.000)	0.050		
**PSA level after BPH surgery, ng/ml**		0.564 (0.356–0.893)	0.015	1.978 (0.668–5.860)	0.218
**PSA density after BPH surgery, ng/ml**^**2**^		0.800 (0.684–0.936)	0.005	0.684 (0.469–0.997)	0.048
**Prostate volume, mL**		1.019 (0.993–1.046)	0.145		
**Resection volume, ml**		1.022 (0.982–1.065)	0.284		
**Suspicious lesion on MRI**					
**Yes**		1 (Ref)		1 (Ref)	
**No**		14.931(4.07954.649)	<0.001	11.827(3.10345.073)	0.001
**Gleason score**					
**6 (ISUP G1)**		1 (Ref)			
**7**	**(ISUP G2)**	Unclear (0.000-Max)	0.999		
	**(ISUP G3)**	1.647 (0.318–8.530)	0.552		
**≥8 (ISUP G4)**		1.176 (0.213–6.505)	0.852		
**Stage**					
**T1a**		1 (Ref)			
**T1b**		0.478 (0.193–1.189)	0.112		

PSA, prostate-specific antigen; BPH, benign prostatic hyperplasia; ISUP, International Society of Urological Pathologists; OR, Odds ratio; CI, confidence interval

### Diagnostic accuracy for pT0 of mpMRI and PSA density after BPH surgery

**Fig 2:**
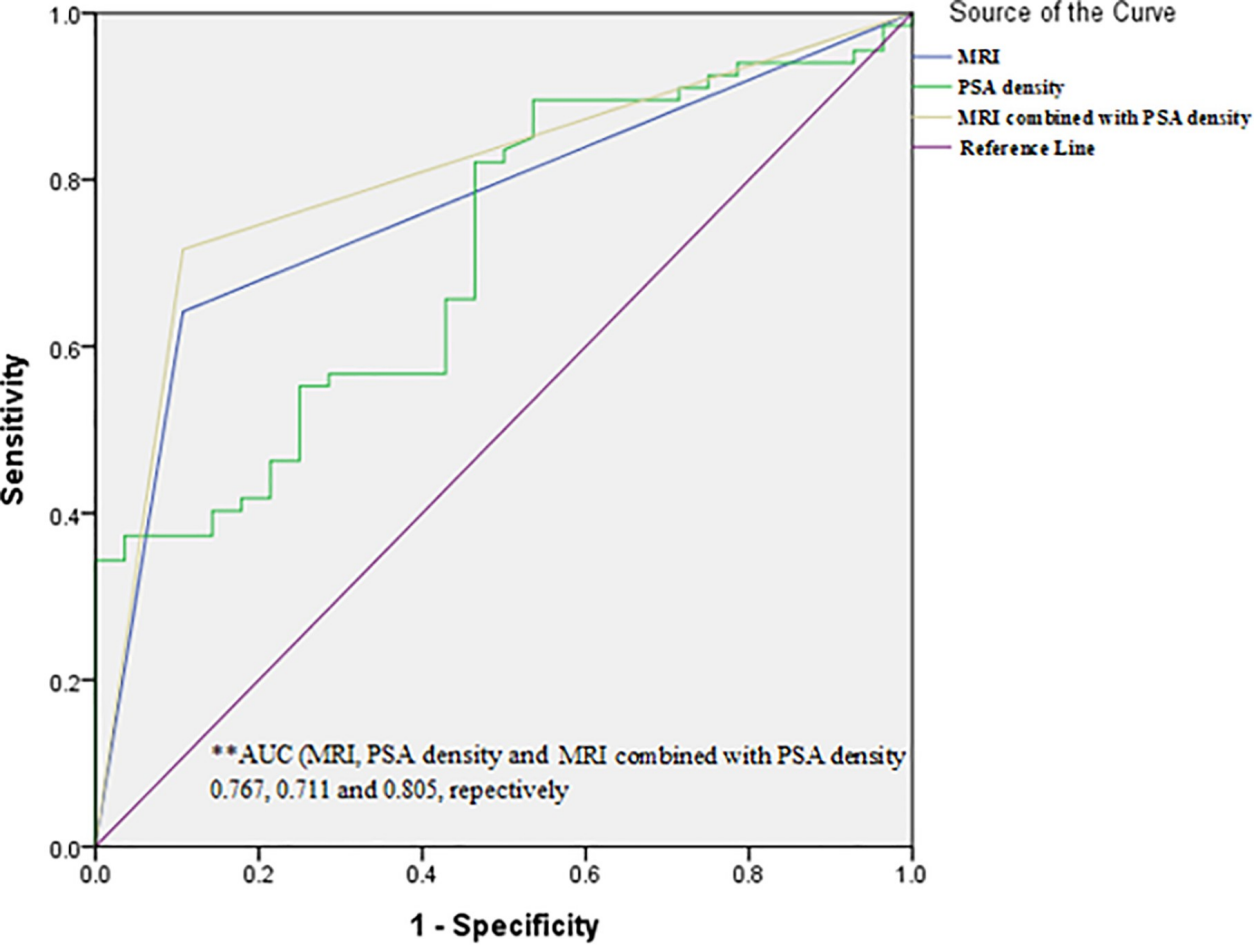
Receiver operating characteristic curves of MRI, PSA density, and MRI combined with PSA density for predicting the presence of residual tumor (pT0).

The AUCs of the ROC curve for mpMRI and PSA density after BPH surgery were 0.767 (95% CI, 0.668–0.867) and 0.711 (95% CI, 0.602–0.820), respectively. Diagnosis of pT0 with mpMRI alone revealed sensitivity of 64.2% (95% CI, 51.5–75.5), specificity of 89.3% (95% CI, 71.8–97.7), PPV of 93.5% (95% CI, 82.9–97.7), and NPV of 51.0% (95% CI, 42.4–59.5). We enhanced the diagnostic accuracy of mpMRI by adding PSA density values. Notably, PSA density ≤0.08 showed the best correction value. The AUC of the ROC curve for this combination of predictive factors was 0.805 (95% CI, 0.711–0.879). Diagnosis of pT0 with the combined predictors revealed sensitivity of 71.7% (95% CI, 59.3–82.0), specificity of 89.3% (95% CI, 71.8–97.7), PPV of 94.1% (95% CI, 84.4–97.9) and NPV of 56.8% (95% CI, 46.8–66.3) (Fig 2) (Table 4).

**Table 4:** Diagnostic accuracy for pT0 of mpMRI and PSA density after BPH surgery.

**A**										
			**Residual tumor**				**No residual tumor (pT0)**			
**PSA density**	≤0.08		44 cases				28 cases			
	>0.08		23 cases				0 cases			
**Suspicious lesion on MRI**	Yes		43 cases				3 cases			
	No		24 cases				25 cases			
**Suspicious lesion on MRI combined with PSA density (≤0.08)**	Yes		48 cases				3 cases			
	No		19 cases				25 cases			
**B**										
**Diagnostic Accuracy for pT0**	**Sensitivity**	**95% CI**		**Specificity**	**95% CI**	**PPV**		**95% CI**	**NPV**	**95% CI**
**mpMRI alone**	64.18	51.5–75.5		89.29	71.8–97.7	93.5		82.9–97.7	51	42.4–59.5
**mpMRI combined with PSA density (≤0.08)**	71.64	59.3–82.0		89.29	71.8–97.7	94.1		84.4–97.9	56.8	46.8–66.3

PSA, prostate-specific antigen; BPH, benign prostatic hyperplasia; PPV, positive predictive value, NPV, negative predictive value; CI, confidence interval

## Discussion

pT0 is a well-known rare phenomenon, occurring in <1% of all patients who undergo RP for PCa [19]. This is strongly associated with neoadjuvant therapy [20]. However, pT0 after RP in IPCa exhibits a different incidence pattern; pT0 in IPCa patients without neoadjuvant therapy should be considered as complete resection of cancer by initial surgery for BPH treatment.

Several studies have reported the prevalence of pT0 cases in patients with T1a–T1b treated with RP; these range from 2% to 48% in patients without previous neoadjuvant therapy [5,8–10,21]. However, there have been few studies to predict pT0. A study regarding prediction of residual tumors by Capitanio et al. [11] was published in 2010. Of 158 cases, T0 was found in 22; PSA before and after BPH operation was significant associated with pT0. However, univariate statistics were used in that study univariate; multivariate analyses were not performed. Similar to that study, postoperative PSA was a significant factor in univariate analyses in our study. In multivariate statistics, however, it was not significant. Therefore, we included PSA density before and after BPH surgery [22–24]; we found that the PSA density was a significant predictor of pT0 in IPCa. In our study, PSA density after BPH surgery was significant both in univariate and multivariate analyses. In addition, our prediction of pT0 was based on mpMRI performed after BPH surgery. To standardize the evaluation and reporting of prostate MRI, the European Society of Urogenital Radiology published guidelines based on an expert consensus in 2012 (PI-RADS). These guidelines were updated to PI-RADS version 2 in 2015 [13,14]. Notably, there is a growing role for mpMRI in the diagnosis of PCa. Therefore, we used a significant lesion on mpMRI as a predictor of pT0 in patients with IPCa; this yielded statistically significant results in our study. However, in many studies, a clinically significant lesion is present, although it remains invisible on mpMRI. Therefore, mpMRI is not a definite factor for determining the presence of residual tumor. In our study, 49 patients (51.58%) with IPCa exhibited invisible lesions on mpMRI; 24 of them had residual cancer after RP. Of these 24 patients, 6 exhibited G7, 2 exhibited T3 pathology, and 2 exhibited BCR during the follow-up period. Therefore, IPCa patients with invisible lesion on mpMRI may exhibit a clinically significant oncologic outcome. We believe it is difficult to use mpMRI alone as a method to predict pT0; thus, we added PSA density after BPH surgery, which was significantly associated with pT0, to increase the diagnostic accuracy of our predictive approach. When mpMRI and PSA density after BPH surgery were combined, the diagnostic accuracy of pT0 was improved, compared with mpMRI alone. We believe this may constitute a good diagnostic method for prediction of pT0. However, despite the use of this diagnostic method, residual cancer after RP was found 3 patients. Therefore, in patients with IPCa who have an invisible lesion on mpMRI, and who exhibit PSA density ≤0.08 after BPH surgery, if no operation is performed, active surveillance is necessary. In addition, biopsy of suspicious lesions on mpMRI after BPH surgery may be a good choice.

Our study has several limitations. First, this was a retrospective review of data from patients treated at a single institution; therefore, multi-center, prospective studies are needed. If we conduct a multi-center study and collect more cases, we may be able to generate a nomogram for residual tumors in patients with IPCa [25]. Second, prostate biopsy was performed simultaneously with BPH surgery for 35 patients enrolled this study. Therefore, there were images of prostate biopsy hemorrhage in postoperative mpMRI. This might have caused the image quality to deteriorate [26]. However, we consulted with radiologists and confirmed that this did not limit interpretation of the image [27]. From 2006 to 2009, patients did not undergo DWI and ADC as part of mpMRI. In consultation with radiologists at our institution, we reviewed suspicious lesions and re-read them on the basis of PIRADS^V2^ recommendations. There were 38 patients from 2006 to 2009, and 57 patients from 2010 to the end of the study period. There was no significant difference with regard to suspicious lesions in the accuracy of mpMRI between the two groups (Group 1 from 2006 to 2009: AUC 0.764, Group 2 beyond 2010: AUC 0.774).

Despite these limitations, our study remains informative for clinicians who treat patients with IPCa. With regard to the strengths of our study, to the best of our knowledge, this is the first investigation to include mpMRI of IPCa for prediction of pT0. Furthermore, we investigated long-term follow up oncologic outcomes of IPCa with an established protocol. No patients were treated with adjuvant androgen deprivation therapy or radiotherapy until BCR; this allowed us to observe the natural history of BCR after RP. In this study, we determined the oncologic outcome according to the presence of residual tumor in IPCa. Because the oncologic outcome of IPCa patients who exhibit pT0 is good, it is important to predict pT0 where possible. Therefore, we believe that the proposed diagnostic tool is a method to reduce overtreatment in pT0 patients. We believe that our study will help clinicians to determine the direction of treatment in IPCa.

## Conclusions

Our results demonstrate that patients with pT0 in IPCa showed a good prognosis; therefore, radical treatment may constitute overtreatment. In our study, lesions invisible on mpMRI, combined with PSA density after BPH surgery, were significantly associated with pT0. Therefore, in IPCa, patients with PSA density ≤0.08 after BPH surgery and with invisible cancer lesion on mpMRI should be considered for active surveillance.
